# Applicability of Bolton's Analysis: A Study on Jaipur Population

**DOI:** 10.5005/jp-journals-10005-1147

**Published:** 2012-08-08

**Authors:** Mridula Trehan, Sonahita Agarwal, Sunil Sharma

**Affiliations:** Professor and Head, Department of Orthodontics and Dentofacial Orthopedics, Mahatma Gandhi Dental College and Hospital, Jaipur, Rajasthan, India, e-mail: mridula_jaipur@yahoo.com; Senior Lecturer, Department of Orthodontics and Dentofacial Orthopedics, Mahatma Gandhi Dental College and Hospital, Jaipur Rajasthan, India; Principal, Professor and Head, Department of Oral and Maxillofacial Surgery, Mahatma Gandhi Dental College and Hospital, Jaipur Rajasthan, India

**Keywords:** Bolton's analysis, Mesiodistal widths

## Abstract

This study was undertaken to compare the Bolton's anterior and overall ratios among males and females in Jaipur population. One hundred study models (50 males and 50 females) of orthodontic patients were randomly selected from the Department of Orthodontics, Mahatma Gandhi Dental College, Jaipur. The normative data for the mesiodistal widths of males and females were established and the anterior and overall ratios were obtained for both males and females. It was observed that were no significant differences in both the ratios in males and females. Hence, both the ratios were obtained for the sample as a whole. These ratios were found to be similar to the Boltons standard with no significant differences. Therefore, it can be concluded that Boltons standards can be applied to this population irrespective of sex or ethnic background.

**How to cite this article:** Trehan M, Agarwal S, Sharma S. Applicability of Bolton's Analysis: A Study on Jaipur Population. Int J Clin Pediatr Dent 2012;5(2):113-117.

## INTRODUCTION

The ratio of maxillary and mandibular tooth sizes represent an important aspect in the diagnosis and treatment planning of an individual. Any discrepancy in the tooth size ratios can dictate the treatment plan as to whether extractions are required or reproximation can suffice. A good posttreatment occlusion depends on an appropriate relationship of the maxillary and mandibular teeth.

Because different tooth sizes have been associated with ethnic groups, it is logical to expect that differences in tooth widths can directly affect tooth-widths ratios.^1^

Bolton developed his overall and anterior ratios based on 55 patients with excellent class I occlusions.^2^ Although Bolton's analysis has proven extremely useful in the clinical setting to guide the orthodontist in cases with extreme tooth size discrepancies, it is not without limitations. Firstly, as Bolton's study included only cases with excellent occlusion, its feasibility in different malocclusions is questionable. Secondly, and perhaps more important, since gender composition of Bolton's sample was not specified, it implies potential selection bias.^3^ Also, most of the cases taken up in his study were orthodontically treated (nonextraction) but the methods of gaining space have not been specified.^2^

The dental literature is replete with studies comparing tooth size discrepancy and malocclusion in different ethnic groups. However, only a few of them included sexual dimorphism and additional data are necessary to understand this relationship.^4^

As in many other human attributes, teeth vary in size between males and females. Gender differences have been reported in the literature and may have clinical relevance. Male teeth are generally recognized to be larger than female teeth. There is also a lack of agreement regarding gender differences in relation to the tooth size proportion between upper and lower anteriors.^5^

As tooth size discrepancies tend to be population specific, this study was taken up in Jaipur population to assess the applicability of Bolton's ratios to this population.

The purposes of the study were the following:

1. Establish normative data on the mesiodistal crown dimensions of the permanent dentition in Jaipur population.
2. Compare both the anterior and overall tooth size ratios of Jaipur population to the ratios available from the Bolton's study.

## MATERIALS AND METHODS

The sample consisted of study models of randomly selected 100 orthodontic patients ranging in ages from 15 to 21 years from the Department of Orthodontics, Mahatma Gandhi Dental College, Jaipur. The subjects were divided into 50 males and females each.

The selection criteria were as follows:

1. All permanent teeth present in each arch (excluding third molars) and in a sufficient state of eruption
2. Good-quality study casts
3. Absence of mesiodistal and occlusal abrasions, caries or class II restorations
4. Absence of dental prosthesis
5. Absence of tooth anomalies such as in form, structure, and development
6. No previous or ongoing orthodontic treatment.

The following rejection criteria were used in selecting groups:

1. Gross restorations, buildups, crowns, onlays, class II amalgams or composite restorations that affect the tooth's mesiodistal diameter
2. Congenital defects or deformed teeth
3. Obvious interproximal or occlusal wear of teeth.

**Fig. 1:**
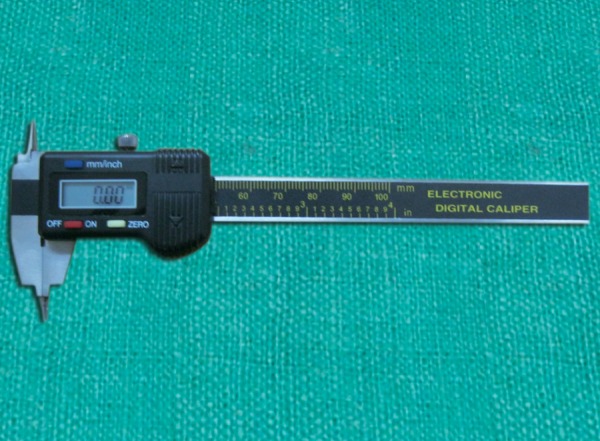
The digital dental calipers used in the study

A pair of digital dental calipers (0-300 mm, Masel Ortho, UK) with a resolution of 0.01 mm, accuracy of ± 0.02 mm/0/0.001'' and a repeatability of 0.01mm/0/0.0005'' was used to measure mesiodistal diameter of each tooth to the nearest 0.01 mm (Fig. 1).

For the main study, the primary investigator measured each tooth twice, from the right first molar to the left first molar in each arch. If the difference was less than 0.2 mm, the first measurement was registered. If the second measurement differed by more than 0.2 mm from the first, the tooth was measured again, and only the new measure was registered. Only 8 to 10 pairs of models were measured each day to prevent visual fatigue.

**Fig. 2:**
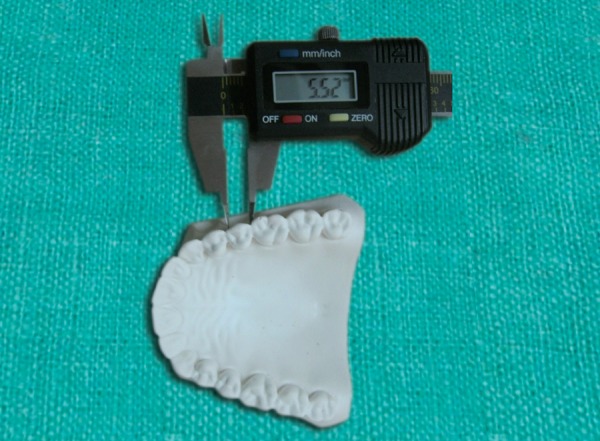
Measurement of the mesiodistal width of the tooth using the digital dental calipers

The width of each tooth was measured from its mesial contact point to its distal contact point at its greatest interproximal distance (Fig. 2). Boltons anterior (canine to the canine) and overall (first molar to first molar) ratios were calculated with the following formulas.

(Sum mandibular 12 / Sum maxillary 12) × 100 = overall ratio (%)

(Sum mandibular 6 / Sum maxillary 6) × 100 = anterior ratio (%)

The t-test for independent groups was used to determine statistical differences between sexes.

## RESULTS

**Graph 1:**
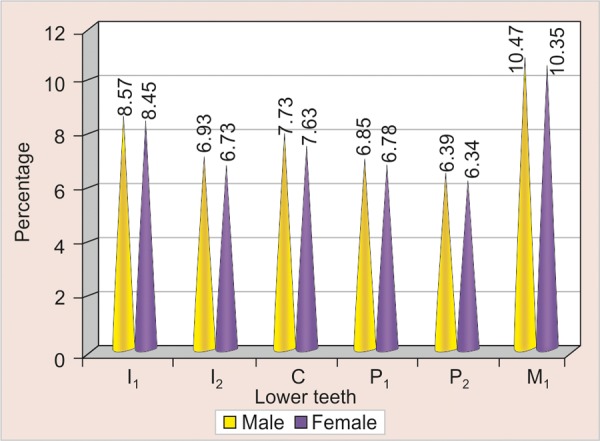
Mean of individual teeth in the upper arch in males and females (I_1_: Central incisor; I_2_: Lateral incisor; C: Canine; P_1_: First premolar; P_2_: Second premolar; M_1_: First molar)

The results are summarized as below:

Table 1 compares the mean values of the mesiodistal widths of individual teeth in males and females in the upper arch. It was observed that in the upper arch there was no significant difference in the mesiodistal widths of any tooth irrespective of the sex (Graph 1).

**Graph 2:**
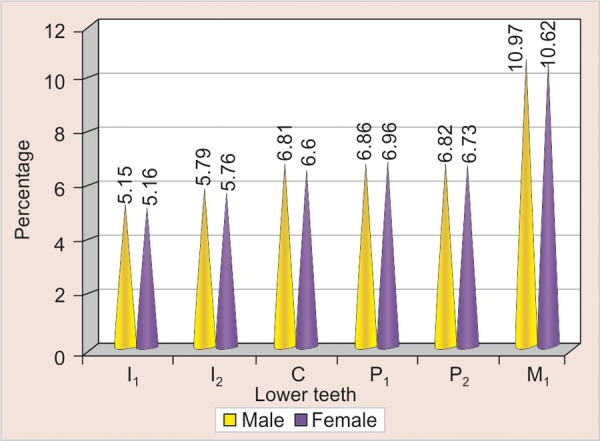
Mean of individual teeth in the lower arch in males and females (I_1_: Central incisor; I_2_: Lateral incisor; C: Canine_;_ P_1_: First premolar; P_2_: Second premolar; M_1_: First molar)

Table 2 compares the mean values of the sum of mesiodistal widths of the individual teeth in males and females separately in the lower arch. Similar results were obtained in lower arch also with no significant difference in males and females (Graph 2).

It was concluded that the sum of the mesiodistal widths of in males (92.96 mm, 84.8 mm) were slightly more than that of females (91.69 mm, 83.65 mm) teeth in both the arches. However, this difference was statistically not significant both in the upper and lower arches (p-value > 0.05) (Table 3).

**Graph 3:**
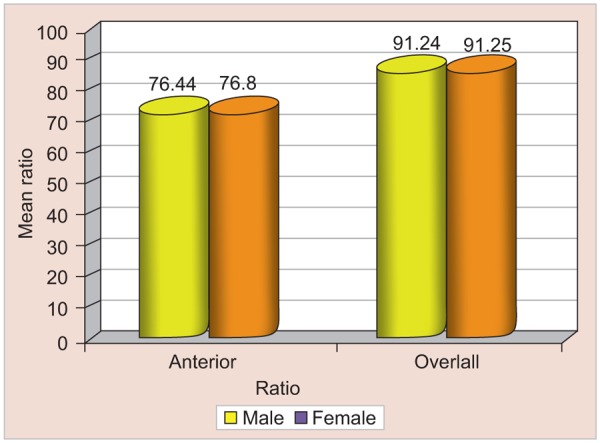
Anterior and overall tooth width ratios in males and females

The mean, standard deviation and p-values for maxillary to mandibular tooth width ratios are given in Table 4 (Graph 3). Although the anterior ratio and overall ratio were slightly higher in females than in males the difference was not significant (p-value >0.05).

**Table 1:** **Table 1:** Mean, standard deviation and p-value of individual teeth in the upper arch

*Upper*		*Mean ± SD*				*p-value*		*Significance*	
		*Male*		*Female*					
I_1_		8.57 ± 0.52		8.45 ± 0.55		>0.05		NS	
I_2_		6.93 ± 0.53		6.73 ± 0.58		>0.05		NS	
C		7.73 ± 0.38		7.63 ± 0.42		>0.05		NS	
P_1_		6.85 ± 0.40		6.78 ± 0.48		>0.05		NS	
P_2_		6.39 ± 0.42		6.34 ± 0.43		>0.05		NS	
M_1_		10.47 ± 0.53		10.35 ± 0.52		>0.05		NS	

I_1_: Central incisor; I_2_: Lateral incisor; C: Canine, P_1_: First premolar; P_2_: Second premolar; M_1_: First molar; NS: Not significant

**Graph 4:**
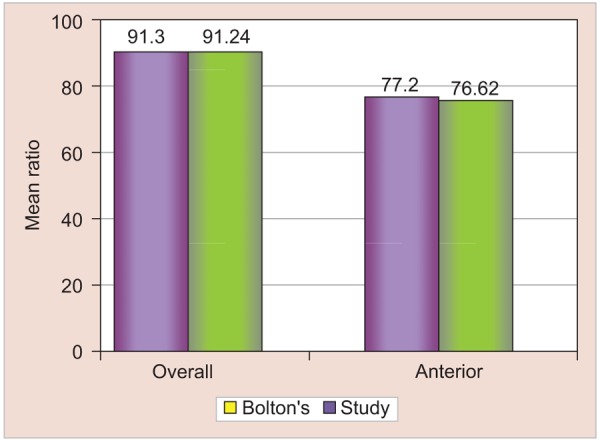
Anterior and overall tooth width ratios compared to the Bolton's ratios

Therefore, combined male and female anterior and overall ratio was derived and compared with that of the Boltons standards (Table 5 and Graph 4).

It was found that the mean differences between the ratios derived in this study were comparable to that of the standards set by Bolton and the mean difference between them was not significant with respect to both the anterior and overall ratio (p-value >0.05).

**Table 2:** **Table 2:** Mean, standard deviation and p-value of individual teeth in the lower arch

*Lower*		*Mean ± SD*				*p-value*		*Significance*	
		*Male*		*Female*					
I_1_		5.15 ± 0.58		5.16 ± 0.42		>0.05		NS	
I_2_		5.79 ± 0.43		5.76 ± 0.51		>0.05		NS	
C		6.81 ± 0.41		6.60 ± 0.44		>0.05		NS	
P_1_		6.86 ± 0.40		6.96 ± 0.44		>0.05		NS	
P_2_		6.82 ± 0.45		6.73 ± 0.45		>0.05		NS	
M_1_		10.97 ± 0.58		10.62 ± 0.64		>0.05		NS	

I_1_: Central incisor; I_2_: Lateral incisor; C: Canine; P_1_: First premolar; P_2_: Second premolar; M_1_: First molar

**Table 3:** **Table 3:** Mean, standard deviation and p-value of teeth in the upper and lower arches

*Group*		*Mean + SD*				*p-value*	
		*Males*		*Females*			
Upper		92.96 ± 4		91.69 ± 4.37		0.13	
Lower		84.80 ± 4		83.65 ± 4.17		0.16	

**Table 4:** **Table 4:** Anterior and overall tooth width ratios in males and females

*Ratio*		*Mean ± SD*				*p-value*		*Significance*	
		*Male*		*Female*					
Anterior		76.44 ± 4.14		76.80 ± 2.91		>0.05		NS	
Overall		91.24 ± 2.87		91.25 ± 2.36		>0.05		NS	

NS: Not significant

**Table 5:** **Table 5:** Anterior and overall tooth width ratios in the whole sample compared with Bolton's ratios

*Ratios*		*Mean ± SD*				*p-value*		*Significance*	
		*Study*		*Bolton*					
Anterior		76.62 ± 3.58		77.20 ± 2.44		>0.05		NS	
Overall		91.24 ± 2.63		91.30 ± 1.71		>0.05		NS	

NS: Not significant

## DISCUSSION

### Mesiodistal Crown Widths

The lower first molar showed the highest variability whereas the upper canine showed the least variability among maxillary and mandibular teeth.

### Sex Differences

In the upper arch the lateral incisor showed maximum variability in females whereas the first molar and the lateral incisors showed maximum variability in males. Maximum variability was seen in the central incisor and first molar in males whereas only first molar had the highest variability in females in the lower arch. The mesiodistal widths of teeth in both the arches followed similar distribution in males and females with the measurements being slightly larger in males although not significant statistically.

This was in concordance with the study done by Santoro et al^6^ on Dominican American population but contrary to the studies done by Lavelle et al^7^ and Moorees et al.^8^ A similar study done on North Indian population observed that teeth tend to be larger in males although the study did not evaluate their significance.^9^ Studies done by Arya et al^10^ and Lavelle et al^7^ showed that there were differences in tooth size between sexes, as reported by a number of authors.

### Anterior and Overall Ratios Compared with Bolton's Study

The anterior and overall ratio were compared between the sexes. It was observed that there was no significant difference in the anterior and overall ratios in either of the sexes. This could be attributed to the similar distribution of the mesiodistal widths of teeth in males and females. This was similar to a study done on Southern Chinese population.^11^ However, Nie and Lin indicated no significant sexual dimorphism for anterior and posterior tooth size ratios in different malocclusion groups. Richardson and Malhotra reported no differences in upper and lower anterior tooth size proportions, indicating that there is a constant 77% ratio for both genders.^12^

As there were no significant differences in anterior and overall ratios between sexes, ratios were obtained for the sample as a whole. These ratios were then compared with the ratios derived from Bolton's study. Interestingly it was observed that the ratios obtained in this study were comparable with that of Bolton's study. The difference between the two was not at all significant. This could be due to the fact that the mean difference between the tooth widths in males and females were not significant and Bolton's study also did not take gender into consideration. This implies that the Bolton's ratios can be applied to this population sample.

## CONCLUSION

The following conclusions have been drawn from the present study:

1. The mesiodistal widths of teeth was marginally greater in males than in females although the difference was statistically not significant. Females showed higher variability than males in the tooth sizes.
2. The mean tooth width ratios, both anterior and overall were similar in males and females with no significant difference between them. Hence, both the ratios were derived for the whole sample and these ratios can be applied irrespective of the gender.

When compared with the standard Bolton's ratios it was found that the ratios derived in this study were comparable to those derived in the Bolton's study. Hence, Bolton's values can also be applied to our population. Evaluation of any intermaxillary discrepancy before final tooth alignment will be beneficial in diagnosis and treatment planning to the clinician and also in meeting the expectations of the patient.
